# The InN epitaxy via controlling In bilayer

**DOI:** 10.1186/1556-276X-9-5

**Published:** 2014-01-06

**Authors:** Jin Zhou, Qiangcan Huang, Jinchai Li, Duanjun Cai, Junyong Kang

**Affiliations:** 1 Fujian Key Laboratory of Semiconductor Materials and Applications, Department of Physics, Xiamen University, Xiamen 361005, People’s Republic of China

**Keywords:** InN epitaxy, MOVPE, Bilayer control, Penetrated nitridation

## Abstract

The method of In bilayer pre-deposition and penetrated nitridation had been proposed, which had been proven to have many advantages theoretically. To study the growth behavior of this method experimentally, various pulse times of trimethylindium supply were used to get the optimal indium bilayer controlling by metalorganic vapour phase epitaxy. The results revealed that the InN film quality became better as the thickness of the top indium atomic layers was close to bilayer. A following tuning of nitridation process enhanced the quality of InN film further, which means that a moderate, stable, and slow nitridation process by NH3 flow also plays the key role in growing better-quality InN film. Meanwhile, the biaxial strain of InN film was gradually relaxing when the flatness was increasingly improved.

## Background

The unique properties of InN are currently attracting much interest in the research community [1,2]. Because of its lowest effective mass and the highest electron drift velocity among all III-nitride semiconductors [3], InN is promising for high-speed and high-frequency electronic devices. And recently, the band gap of InN, which is considered as 1.9 eV, is renewed to approximately 0.7 eV [4-6], covering a broad range of wavelength from near infrared at approximately 1.5 μm to ultraviolet at approximately 200 nm based on its direct band gap alloying with GaN and AlN [7-9]. However, the achievement of high crystalline quality InN film is still a great challenge, which attributed to the lack of lattice-matched substrate, the low dissociation temperature (approximately 600°C), the low pyrolysis efficiency of NH_3_ at 500 to 600°C, the pre-reaction of the precursors before arriving at the substrate surface, and also the InN clustering effect [10-14].

In order to achieve high-quality InN film, effort has been made by researchers with different methods such as optimizing growth temperature, controlling V/III ratio, introducing buffer layer, or employing pulsed atomic layer epitaxy technique [15,16]. However, the crystalline quality of InN film is still far below a satisfactory level due to the existence of huge quantity of defects [16]. To elucidate the original difficulty in In film deposition, the formation kinetics of InN with N and In atoms on the In polar GaN surface has been systematically studied by first-principles calculations [17], it was found that the pre-deposition of In bilayer on the surface could improve the In migration on the surface and the smoothness of In film.

In this work, the epitaxy method of In bilayer controlling and penetrated nitridation was employed for the InN film growth on GaN template. In order to determine critical trimethylindium (TMI) flow required for forming In bilayer, the pulse time of TMI supply was optimized. The results revealed that the film quality became better as the thickness of the top indium atomic layers was close to bilayer. Based on the In bilayer deposition, a moderate, stable, and slow nitridation process by NH3 flow also played the key role in growing better-quality InN film. X-ray diffraction (XRD) measurements confirmed the gradual relaxation of biaxial strain in InN epilayers during increment of the smoothness.

## Methods

### Growth of samples

**Figure 1:**
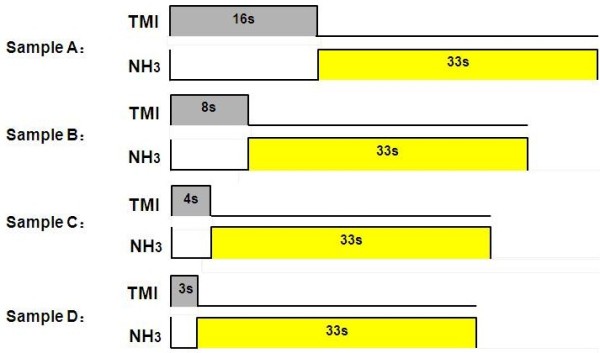
The pulse timing's schematic diagrams of samples A to D.

InN films were grown on a 3-μm-thick GaN template with(0001) sapphire substrate by using metalorganic chemical vapor deposition (MOCVD) system with a Thomas Swan closely coupled showerhead (CCS) reactor. The trimethylgallium (TMG), trimethylindium (TMI), and ammonia (NH_3_) were used as the precursors for Ga, In, and N, respectively, and H_2_ and N_2_ were used as the carrier gasses. Prior to the GaN/AlGaN superlattice growth, thermal cleaning of the (0001)-oriented sapphire substrate was carried out under hydrogen ambient at 1,050°C for 10 min to remove native oxide from the surface. Then, an approximately 30-nm low-temperature GaN buffer layer (approximately 570°C) was grown followed by a approximately 3-μm high-quality GaN underlaying layer (approximately 1,090°C). During the stage of InN growth, the pressure was set to 450 torr at 550°C [18]. In order to accurately control the deposition of indium atomic multilayers and the following nitridation process, the pulse growth method was employed through switching and adjusting the pulsed supply time of TMI and ammonia flows, as shown in Figure 1. For samples A, B, C, and D, a constant TMI flow of 2.0 × 10^−5^ mol/min was used whereas a series of duration time of the pulsed TMI flow, 16, 8, 4, and 3 s, was applied, respectively. Then, they were followed by a 33-s pulse of NH_3_ flow for the nitridation process. The mole flow of ammonia was set to be 0.5 mol/min. For the purpose of comparison, the total moles of indium supply in different samples was preserved by varying the total pulse periods of 180, 360, 720, and 960 for samples A to D, respectively. In order to avoid the possibility of over-nitridation, a lower ammonia flow of 0.25 and 0.125 mol/min was set for samples E and F, respectively, and other growth parameters were as the same as those set for sample C.

### Characterization

The thickness of film was measured by *in situ* growth monitoring curves (Panalytical X'pert PRO X, Panalytical, Almelo, The Netherlands). The surface morphology and smoothness of the as-grown samples were characterized by atomic force microscopy (AFM, PicoSPM and PSI XE-100, Molecular Imaging, Ann Arbor, MI, USA) and scanning electron microscopy (SEM, LEO 1530, LEO Elektronenmikroskopie GmbH, Oberkochen, Germany) equipped with an energy-dispersive X-ray spectrometer (EDX). The structural quality and the In composition of InN films were evaluated by X-ray diffraction (XRD) in a X’ Pert PRO system.

## Results and discussion

**Figure 2:**
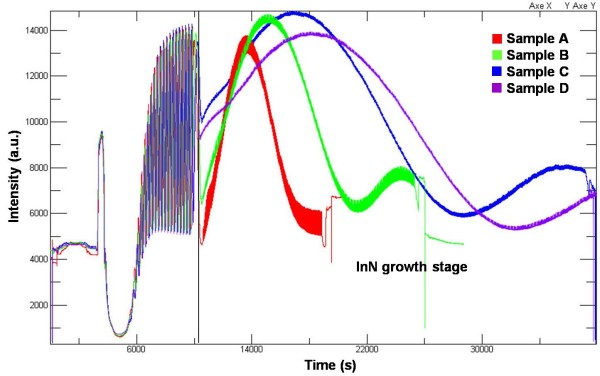
**The *in situ *** **growth monitor interferometery curves of samples A to D.**

**Figure 3:**
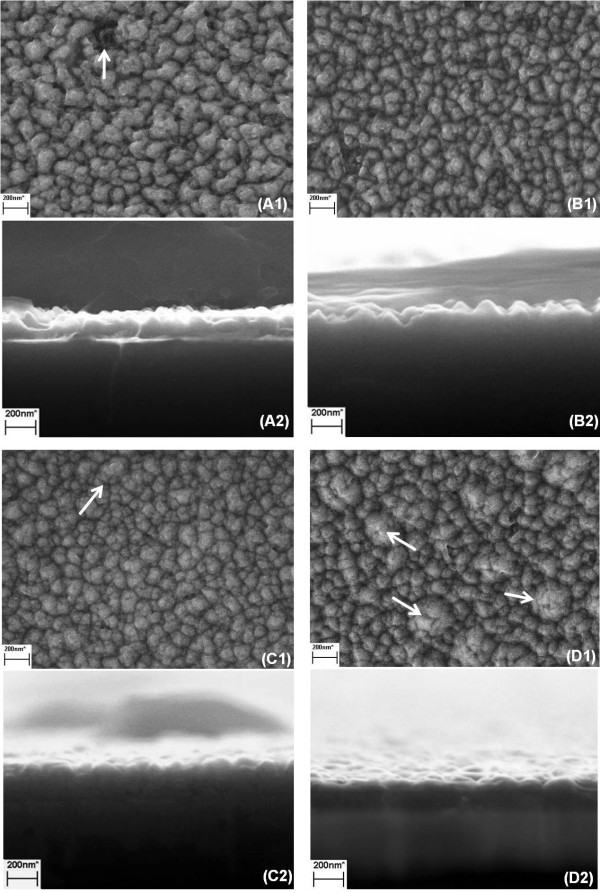
**SEM images of samples A to D. (A1, B1, C1 D1)** The top view and **(A2, B2, C2, D2)** the side view images of samples **A** to **D**, respectively.

In the ideal indium bilayer construction process, we need to deposit one indium monolayer in each pulse, thus this new indium monolayer would construct an indium bilayer with the top indium monolayer which we had deposited in last pulse [17]. Figure 2 shows the *in situ* growth monitoring interferometer curves of samples A to D. One can observe that in the InN growth stage, the vibration of all four sample's curves have experienced nearly equal phase shift. According to this phase shift, we can easily calculate the average InN film's thickness of them, which is about 170 nm. Also this result has been confirmed by practical measurement in the SEM cross-sectional observation (see SEM cross-sectional photos in Figure 3). Thus, the InN deposition thicknesses per period of samples A, B, C, and D are about 9.5, 4.7, 2.4, and 1.8 Å, respectively. According to the (0001) lattice constant *c* of wurtzite InN, the thickness of one In-N monolayer (*c*/*2*) is about 2.8 Å. Comparing to this value, sample C's growth thickness of each pulse is the closest one.

Figure 3 shows the SEM images of surface morphology and cross sections of samples A to D. From the top view of sample A (A1), one can see some obvious dark holes on the surface, indicating the formation of vacancies due to In accumulation in droplets. The formation of holes and droplets easily leads to a pretty rough surface (rms = 33, from AFM scanning result), as shown in Figure 3A2. As we know, the melting temperature of metal In is only about 157°C. Thus, under the growth temperature of InN (550°C), the pulsed deposition of In for a long duration time may form a thick liquid In layer on the surface. By the effect of surface tension, In droplets in large size would come into being quickly. This is the main reason governing the surface roughness. In order to reduce the roughness, the pulse time of TMI is reduced to 8 s for sample B. The obtained InN film shows better flatness (rms = 20) and dark holes have been well removed (Figure 3B2). According to the theoretical simulation of the kinetics of InN formation [17], if the thickness of indium film is larger than two atomic layers, the nitridation of this In film could not well form a InN epilayers in correct stoichiometric ratio (1:1) and the excessive In will lead to roughness. Thus, the TMI pulse time was further decreased down to 4 s. As shown in Figure 3C1, the islands of sample C begin to show regular shape relatively and the surface becomes more flat (rms = 14). Meanwhile, it can be observed that there are some islands in larger size, as indicated by arrow. The number of these types of large islands further increases in sample D (Figure 3D1), in which the TMI pulse time was set to 3 s. This trend of quality deterioration implies that the indium film deposited during the TMI period turns to be less than one atomic layer and fail to construct indium bilayer. This insufficient coverage of indium layer could not provide the advantage of nitridation of indium bi-layer structure. On the contrary, over-nitridation under N-rich condition leads to the deterioration of the InN film quality of sample D. Therefore, it could be determined that 4-s pulsed supply of TMI in sample C is the optimal setting.

**Figure 4:**
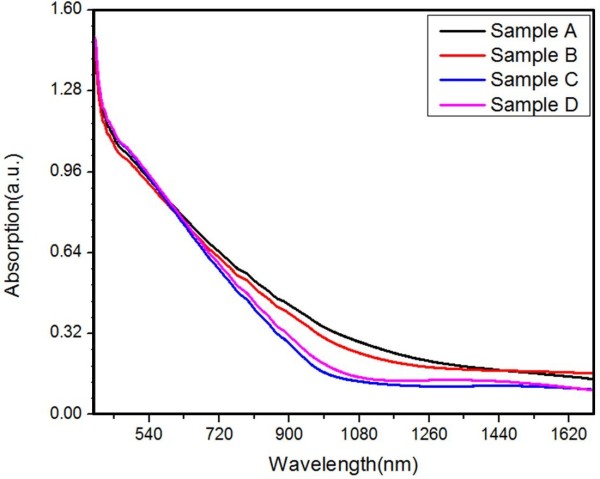
The absorption spectra of samples A to D.

To investigate the optical property of these samples, absorption spectra were recorded to determine the band gap of InN film and the results are shown in Figure 4. Although all four samples' absorption curves show limited differences due to the small thickness or relatively low crystalline quality of the InN film, their differences of slope's changes still can be identified. The absorption spectra of sample C and D have a clear slope threshold near the absorption edge. While, for samples A and B such slope threshold is absent and, beyond 1,100 nm, absorptions related with defect or impurity bands appear. This indicates that sample C has the best film quality due to the optimized pulsed growth with TMI supply. In principle, InN is a direct band semiconductor so that the relationship between its energy band gap and its absorption coefficient could follow the formula below:

(1) $${\left(\mathrm{\alpha hv}\right)}^{2}=k\left(\mathrm{hv}-E_{g}\right),$$

where the *α* is the absorption coefficient and the *E*_*g*_ is the band gap. Thus, the *E*_*g*_ of our samples could be estimated through the intersectional point of absorption edge's tangent and horizontal axis. It is found that the *E*_*g*_ of sample C and D is about 1.22 and 1.19 eV, respectively. Due to the unclear slope thresholds in samples A and B, the *E*_*g*_ is difficult to determine precisely. The range of reasonable *E*_*g*_ for samples A and B would be between 0.7 to 0.9 eV, which is lower than those of sample C and D. This may stem from the absorption associated with the deep level defects and impurities rather than the band edge absorption of InN, the *E*_*g*_ of which should be about 1.25 eV [19].

**Figure 5:**
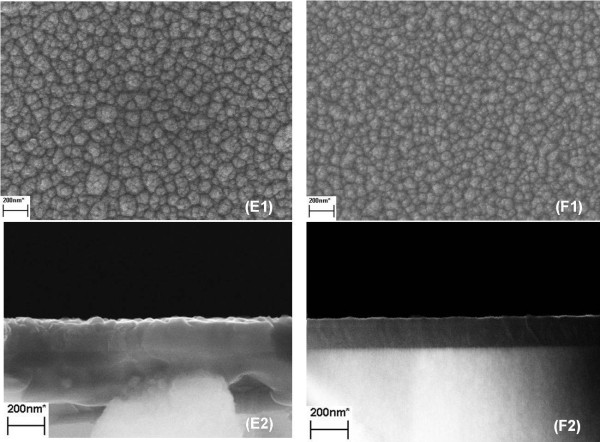
**SEM images of sample E and F. (E1, F1)** The top view and **(E2, F2)** the side view images of samples **E** and **F**, respectively.

Considering the negative influence by the excessive NH_3_ supply, we tried to improve the nitridation process by optimizing the ammonia flow. In principle, the indium bilayer will experience a nitridation process with the penetration of N atoms into between the bilayer [17]. This process would finally form a uniform wurtzite InN structure on the surface. For the case of excessive NH3 flow, the top layer in high N concentration on the surface easily forms a steep concentration gradient between surface and sub-surface layers where the N atoms will diffuse to. According to Fick' first law,

(2) $$J=-D\frac{\partial C}{\partial X},$$

where the *J* is the total diffusion flux and the *D* is the diffusion factor. The steeper the concentration gradient $\left(-\frac{\partial C}{\partial X}\right)$ would lead to the higher the total diffusion flux *J*[20]. Thus, N atoms could not uniquely arrive at the preferable top site via the one-atom-on-one-site mode. Instead, they would diffuse to various positions and some would even crowd in some energy minima. Meanwhile, ultra-high N concentration on surface could even make some N atoms hang over the top indium atomic layer, and, in this case, the indium pre-deposition of next pulse would fail to construct indium bilayer in some regions. As a result, the uniformity and smoothness of the InN film is deteriorated. Based on this analysis, the NH3 flow should be optimized by reducing the mass flow, which is set to 0.25 mol/min for sample E and 0.125 mol/min for sample F. Figure 5 shows the SEM images of these two samples. One can see that the smoothness of sample E has been slightly improved and is better than that of sample C. This indicates that the lower ammonia flow could improve the uniform diffusion of N atoms. Further reduction of NH3 flow in sample F finally leads to a large improvement of InN quality and surface smoothness, as shown in the cross-sectional image of Figure 5F2. The corresponding AFM scanning also confirms this enhancement of surface smoothness (rms = 7). After the deposition of indium bilayer, a moderate, stable, and slow nitridation process with appropriate ammonia flow is crucial for the formation of better-quality InN film.

**Figure 6:**
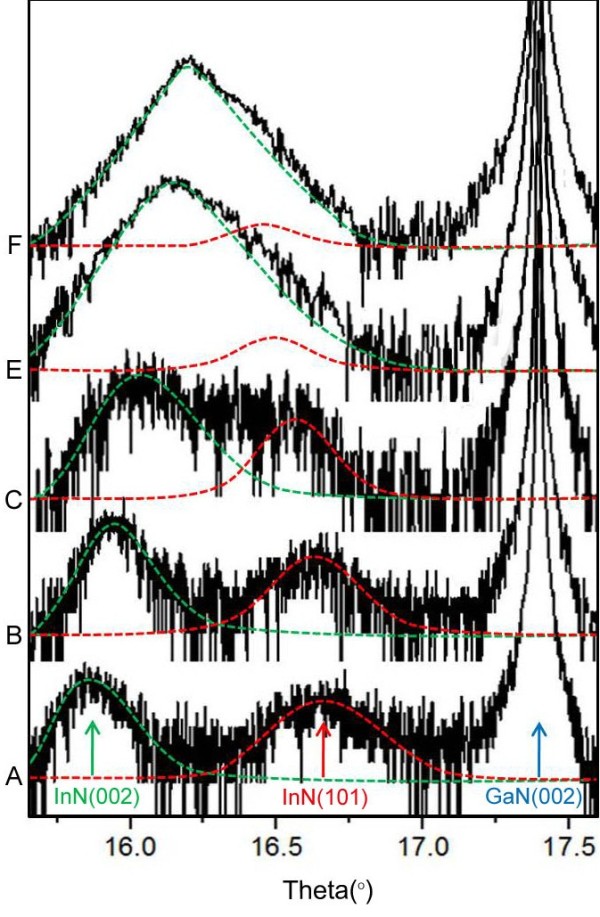
The XRD diffraction spectra of samples A, B, C, E, and F.

In order to study the residual strain of as-grown InN films, XRD characterizations with *ω*-2*θ* scans were taken and the results are shown in Figure 6. Typical symmetrical (002) diffraction peaks of wurtzite InN and wurtzite GaN could be clearly identified, at about 15.8° and 17.4° [21]. Besides, another weak peak was observed at about 16.65°; this peak has been identified as (101) diffraction peak of wurtzite InN by consulting related database and reference. In order to separate the mixing of these two peaks, a multi-peak fitting in this region was made and peak positions of each could be determined. Base on angle of the (002) peaks (green dash lines) and angle of the (101) peaks (red dash lines), the corresponding lattice constant along *c* axis and *a* axis could be obtained simply by using the formula below:

(3) $$d_{\mathrm{hkl}}={\left[\frac{4}{3}\left(\frac{h^{2}+k^{2}+\mathrm{hk}}{a^{2}}\right)+{\left(\frac{l}{c}\right)}^{2}\right]}^{-\frac{1}{2}},$$

where the *d*_*hkl*_ is the interplanar distance between (*hkl*) planes. The complete crystalline data is summarized in Table 1. One can see that the lattice constant *a* is increasing from samples A to F, and the *a* value of sample F (3.63 Å) is very close to the equilibrium value of wurtzite InN (3.627 Å) obtained by first principle calculations, indicating the gradual reduction of residual biaxial strains through growth optimization. Whereas, the (002) peak (correspond to lattice constant *c*) is right shifting correspondingly due to the expansion distortion by the elastic strain on the *a* axis. Meanwhile, it can be seen that the (002) peak is getting dominant, which means a preferential (002) crystal orientation in sample F. All these evidences imply that the biaxial strain has been well relaxed, and the crystal orientation has become better in sample F.

**Table 1:** XRD peak position of (002) diffraction and main lattice constants of InN films for our samples

	**Sample A**	**Sample B**	**Sample C**	**Sample E**	**Sample F**
InN(002) (°)	15.82	15.83	15.95	16.15	16.19
*c*(Å)	5.68	5.67	5.63	5.57	5.56
InN(101) (°)	16.65	16.60	16.53	16.43	16.37
*d101* (Å)	2.70	2.71	2.72	2.73	2.74
*a*(Å)	3.54	3.56	3.58	3.61	3.63

## Conclusions

Through using various pulse times of TMI supply, we achieved optimal indium bilayer control by metalorganic vapour phase epitaxy. When the top indium multilayer was getting close to bilayer, InN film quality had been gradually improved due to high surface migration and good structure consistency of indium bilayer forming. The absorption spectra also confirmed that the InN film which was grown via optimal indium pre-deposited controlling had the fewest defects and impurities. Furthermore, an optimization of ammonia flow during the nitridation stage made an extraordinary improvement of the InN film's flatness; it means that based on the In bilayer controlling deposition, a moderate, stable, and slow nitridation process also plays the key role in growing better-quality InN film. Meanwhile, the biaxial strain of InN film was gradually relaxing when the parameters of growth was optimizing, implying that the mismatch stress of InN heteroepitaxy can be well relaxed via this growth method.

## Competing interests

The authors declare that they have no competing interests.

## Authors’ contributions

JZ carried out the experiments and drafted the manuscript. QCH participated in the preparation and characterization of the samples. JCL participated in the final data analysis and the critical review of the manuscript. DJC and JYK conceived the study, participated in the final data analysis and the critical review of the manuscript. All authors read and approved the final manuscript.
